# The effect of acute exercise on interleukin-6 and hypothalamic–pituitary–adrenal axis responses in patients with coronary artery disease

**DOI:** 10.1038/s41598-020-78286-2

**Published:** 2020-12-07

**Authors:** Zeid Mahmood, Anette Davidsson, Eva Olsson, Per Leanderson, Anna K. Lundberg, Lena Jonasson

**Affiliations:** 1 Unit of Cardiovascular Medicine, Department of Clinical Physiology in Linköping and Department of Health, Medicine and Caring Sciences, Linköping University, Linköping, Sweden; 2 Unit of Clinical Medicine, Occupational and Environmental Medicine Center, and Department of Health, Medicine and Caring Sciences, Linköping University, Linköping, Sweden; 3 Unit of Cardiovascular Medicine, Department of Health, Medicine and Caring Sciences, Linköping University, Linköping, Sweden; 4 Unit of Cardiovascular Medicine, Department of Cardiology in Linköping, and Department of Health, Medicine and Caring Sciences, Linköping University Hospital, Linköping University, 581 85 Linköping, Sweden

**Keywords:** Biomarkers, Cardiology, Endocrinology, Risk factors, Signs and symptoms

## Abstract

Vulnerability to stress-induced inflammation has been linked to a dysfunctional hypothalamus–pituitary–adrenal (HPA) axis. In the present study, patients with known or suspected coronary artery disease (CAD) were assessed with respect to inflammatory and HPA axis response to acute physical exercise. An exercise stress test was combined with SPECT myocardial perfusion imaging. Plasma and saliva samples were collected before and 30 min after exercise. Interleukin (IL)-6 and adrenocorticotropic hormone (ACTH) were measured in plasma, while cortisol was measured in both plasma and saliva. In total, 124 patients were included of whom 29% had a prior history of CAD and/or a myocardial perfusion deficit. The levels of exercise intensity and duration were comparable in CAD and non-CAD patients. However, in CAD patients, IL-6 increased after exercise (p = 0.019) while no differences were seen in HPA axis variables. Conversely, patients without CAD exhibited increased levels of ACTH (p = 0.003) and cortisol (p = 0.004 in plasma, p = 0.006 in saliva), but no change in IL-6. We conclude that the IL-6 response to acute physical exercise is exaggerated in CAD patients and may be out of balance due to HPA axis hypoactivity. It remains to be further investigated whether this imbalance is a potential diagnostic and therapeutic target in CAD.

## Introduction

Inflammation is a major component of atherosclerosis assumed to play a role in both pathogenesis and prognosis of cardiovascular disease^1^. Accumulating evidence indicates that elevations of inflammatory biomarkers predict the risk of developing coronary artery disease (CAD) and myocardial infarction (MI) in healthy individuals^2,3^. In this regard, interleukin 6 (IL-6) and C-reactive protein (CRP), whose production is stimulated by IL-6, have been among the most commonly assayed markers. Moreover, results from two large genetics consortia have provided evidence that the IL-6 signaling pathway has a causal role in the development of CAD^4,5^. IL-6 is also an independent predictor of recurrent cardiovascular events in patients with stable CAD^6^. Importantly, modulation of IL-6 by targeting its upstream mediator IL-1 was recently shown to reduce cardiovascular event rates in CAD patients with previous MI and sustained low-grade inflammation defined as a CRP level of ≥ 2 mg/L^7^.

The sustained inflammatory state in CAD patients does not have one clear underlying cause. It may reflect an ongoing excessive immune response in the arterial wall but also a systemic proinflammatory milieu created by various risk factors, such as obesity, diabetes, smoking and sedentary life style. Overall, there is emerging evidence for an imbalance between pro- and anti-inflammatory actions and a poor control of the inflammatory reaction in atherosclerosis and CAD^1^. In the clinical setting, acute stress (mental or physical) can be used as a method to investigate the ability to control inflammation^8,9^. Interestingly, a few previous studies have shown that patients with CAD display an exaggerated inflammatory response to both mental and physical stress compared with healthy controls, as assessed by CRP or IL-6 elevation in plasma^10,11^. In general, acute exercise has been found to generate a markedly greater IL-6 response than mental stress^10^.

One major regulator of stress response and inflammation is the hypothalamic–pituitary–adrenal (HPA) axis. Normally, stress-induced release of cytokines, like IL-6, activates the HPA axis resulting in rapid increases of adrenocorticotropic hormone (ACTH) and cortisol^12–14^. Cortisol will, as a feedback, suppress further release of cytokines. In various animal models, a blunted HPA axis response has been associated with susceptibility to autoimmune and inflammatory disorders^15^. In human disease, inadequately low cortisol response to stress has been reported in chronic inflammatory processes, such as rheumatoid arthritis, chronic obstructive pulmonary disease and depression^16^. Furthermore, blunted cortisol response to both mental and physical stress has been shown in patients with CAD compared with healthy controls^11,17^. Whether blunted HPA axis response, assessed by ACTH and cortisol release, is linked to stress-induced release of IL-6 in CAD patients has not been previously investigated.

**Figure 1:**
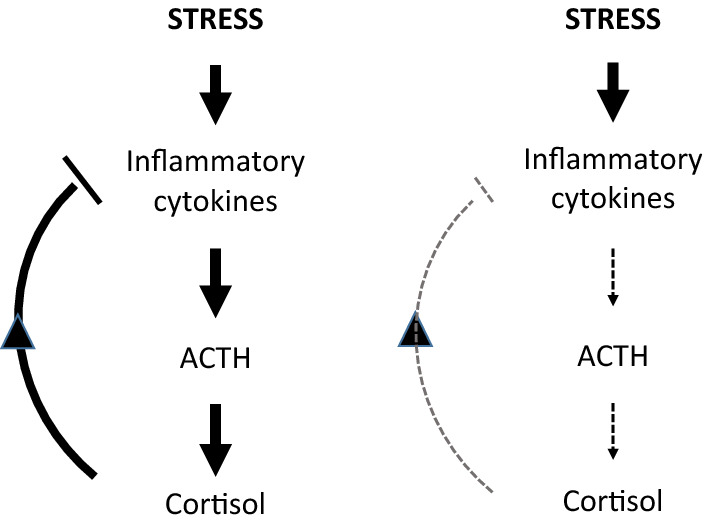
The role of the hypothalamic–pituitary–adrenal (HPA) axis as a regulator of stress response and inflammation. LEFT: Normally, the stress-induced release of cytokines, like IL-6, activates the HPA axis resulting in rapid increases of adrenocorticotropic hormone (ACTH) and cortisol. Cortisol will, as a feedback, suppress further release of cytokines. RIGHT: Hypothetically, a blunted HPA axis response leads to inadequate downregulation of stress-induced inflammatory cytokines.

In the present study, patients with known or suspected CAD were assessed for their ability to control the inflammatory response to stress with focus on the HPA axis response. The hypothesis of inadequate regulation of IL-6 due to blunted HPA axis response is illustrated in Fig. 1. A short bout of exercise on a bicycle ergometer was used as a method to induce a limited inflammatory response. The exercise stress test was combined with SPECT myocardial perfusion imaging (MPI) for the evaluation of exercise-induced ischemia as a potential confounder. The concentrations of IL-6 and ACTH (in plasma) and cortisol (in plasma and saliva) were measured before and after exercise.

## Results

### Patient population and results of exercise stress test

In total, 124 patients were included. Their demographic and clinical characteristics, including medication are shown in Table 1. Thirty patients (24%) had documented CAD, defined by coronary angiography as one or more lesions with > 50% diameter stenosis. Among them, 25 had a history of prior myocardial infarction (MI) and 5 had a history of coronary revascularization (percutaneous coronary intervention or coronary by-pass surgery) due to stable or unstable angina. Results of the exercise stress test and SPECT MPI in all patients are shown in Table 2. At maximal exercise, heart rate and systolic blood pressure showed significant increases (both p < 0.001) compared with baseline. Exercise-induced chest pain was reported by 17 patients (14%). A myocardial perfusion deficit was observed in 19 patients (15%) of whom 4 patients had an irreversible perfusion deficit and 15 patients had an ischemic total perfusion deficit (TPD) ≥ 5%. Among those who reported exercise-induced chest pain, only one exhibited ischemic TPD ≥ 5%.

**Table 1:** Demographic and clinical characteristics, including medication and laboratory variables, of all patients and patients divided into two subgroups: those with documented CAD, defined as a history of prior MI and/or coronary revascularization and/or a myocardial perfusion deficit, and those with no signs of CAD, i.e. no history of prior CAD events and normal SPECT MPI (non-CAD).

	All patients n = 124	CAD n = 36	Non-CAD n = 88	p^a^
Age, years	67 (57–73)	67 (59–73)	66 (56–73)	NS
Female, n (%)	49 (40)	6 (17)	43 (49)	0.001
BMI	27 (24–30)	27 (25–30)	27 (24–29)	NS
Smokers, n (%)	15 (12)	7 (19)	8 (9.1)	NS
Hypertension, n (%)	65 (52)	20 (56)	45 (51)	NS
Diabetes, n (%)	20 (16)	8 (22)	12 (14)	NS
Statin, n (%)	61 (49)	28 (78)	33 (38)	< 0.001
Beta blockers, n (%)	50 (40)	23 (64)	27 (31)	0.002
ACEI/ARB, n (%)	63 (51)	25 (69)	38 (43)	0.029
Calcium channel blockers, n (%)	20 (16)	4 (11)	16 (18)	NS
Low dose aspirin, n (%)	54 (44)	28 (78)	26 (30)	< 0.000
Creatinine, μmol/L	82 (69–96)	85 (70–97)	81 (68–95)	NS
CRP, mg/L	1.0 (0.5–2.6)	0.9 (0.5–1.5)	1.2 (0.5–2.6)	NS

^a^CAD *vs* non-CAD. BMI, body mass index; ACEI/ARB, angiotensin converting enzyme inhibitors/angiotensin receptor blockers; CRP, C-reactive protein.

**Table 2:** Results from exercise stress test and myocardial perfusion imaging of all patients and patients divided into two subgroups: those with documented CAD, defined as a history of prior MI and/or coronary revascularization and/or a myocardial perfusion deficit, and those with no signs of CAD, i.e. no history of prior CAD events and normal SPECT MPI (non-CAD).

		All patients n = 124	CAD n = 36	Non-CAD n = 88	p^a^
Heart rate, beats/min	Baseline	70 (63–80)	68 (63–78)	71 (63–80)	NS
	Maximum	142 (133–155)	138 (133–153	142 (131–155)	NS
Systolic blood pressure, mm Hg	Baseline	138 (125–150)	140 (120–150)	135 (125–150)	NS
	Maximum	190 (180–210)	193 (180–210)	190 (180–210)	NS
Diastolic blood pressure, mm Hg	Baseline	80 (70–88)	80 (70–85)	80 (70–90)	NS
Maximal workload, watts		127 (100–178)	121 (95–184)	130 (102–174)	NS
Exercise duration, min		7.3 (6.2–8.3)	7.3 (6.4–8.6)	7.3 (6.1–8.2)	NS
Left ventricular ejection fraction < 50%, n (%)		10 (8.2)	7 (19)	3 (3.5)	0.007
Exercise-induced chest pain, n (%)		17 (14)	6 (17)	11 (13)	NS
Myocardial perfusion deficit, n (%)		19 (15)	19 (53)	–	
Reversible myocardial perfusion deficit^b^, n (%)		15 (12)	15 (42)	–	

^a^CAD *vs* non-CAD.

^b^Total perfusion deficit, TPD, ≥ 5%.

Biochemical measures before and after exercise are presented in Table 3. All patients showed a significant increase in IL-6, ACTH and cortisol after exercise. Also, fatty acid-binding protein 4 (FABP4) levels increased significantly after exercise. FABP4 was used as a potential surrogate marker of exercise-induced activation of the sympathetic nervous system, as described by Iso et al.^18^.

**Table 3:** Biochemical measures before and after exercise in all patients and patients divided into two subgroups: those with documented CAD, defined as a history of prior MI and/or coronary revascularization and/or a myocardial perfusion deficit, and those with no signs of CAD, i.e. no history of prior CAD events and normal SPECT MPI (non-CAD).

	All patients n = 124	CAD n = 36	Non-CAD n = 88	p^a^
**IL-6, pg/mL**				
Baseline	2.81 (2.17–4.03)	2.90 (2.40–4.01)	2.81 (2.02–4.36)	NS
Exercise	3.11 (2.27–4.33)	3.18 (2.55–4.13)	2.85 (2.07–4.40)	NS
p^a^	0.035	0.019	NS	
**ACTH, pmol/L**				
Baseline	3.13 (2.13–4.33)	3.43 (2.43–5.08)	3.06 (1.96–4.25)	NS
Exercise	3.57 (2.29–5.30)	3.34 (2.01–6.28)	3.59 (2.30–5.18)	NS
p^a^	0.001	NS	0.003	
**Cortisol in plasma, nmol/L**				
Baseline	326 (266–401)	344 (264–415)	319 (264–398)	NS
Exercise	354 (286–443)	339 (286–474)	356 (278–435)	NS
p^a^	< 0.001	NS	0.004	
**Cortisol in saliva, nmol/L**				
Baseline	6.3 (4.0–9.3)	8.2 (3.0–11.3)	6.0 (4.3–8.6)	NS
Exercise	8.2 (6.0–14.8)	7.5 (5.9–20.7)	8.2 (5.9–13.9)	NS
p^a^	0.001	NS	0.006	
**FABP4, pg/mL**				
Baseline	11.3 (7.8–15.9)	10.3 (6.3–15.9)	11.3 (8.1–16.1)	NS
Exercise	12.9 (8.7–17.8)	11.0 (6.9–18.1)	13.2 (9.1–17.3)	NS
p^a^	< 0.001	0.019	< 0.001	

^a^CAD *vs* non-CAD. Baseline vs exercise. IL-6, interleukin-6; ACTH, adrenocorticotrophic hormone; FABP4, fatty acid binding protein-4.

### Patients with or without documented CAD

The patients were divided into two groups: one group with documented CAD and/or the presence of myocardial perfusion deficit (CAD, n = 36) and one group without documented CAD, i.e. no history of prior CAD events and normal SPECT MPI (non-CAD, n = 88). Table 1 shows the descriptive information for CAD and non-CAD patients. There was a dominance of females in the non-CAD group but, otherwise, no differences in variables such as age, BMI, smoking, hypertension, diabetes and plasma levels of creatinine and CRP. Not unexpectedly, the use of cardiovascular medications, including statins, beta-blockers, angiotensin converting enzyme (ACE) inhibitors/angiotensin receptor blockers and low-dose aspirin (75 mg daily) was lower in the non-CAD group, while the use of anti-depressive agents did not differ (CAD 8.3%, non-CAD 11%). Results of the exercise stress tests and SPECT MPI in the CAD and non-CAD groups are also given in Table 2. Heart rate and blood pressure at rest and maximal exercise were similar in the two groups, and so was exertion level, defined by maximal watt load and exercise duration. The prevalence of exercise-induced chest pain was also similar in CAD and non-CAD patients. There was a higher proportion of individuals with reduced left ventricular ejection fraction (< 50%) in the CAD group.

In the non-CAD group, the indications for SPECT MPI were in the majority of cases atypical chest pain or dyspnea and inconclusive results of exercise test. Other indications included pathological electrocardiogram (ECG) findings and family history of premature CAD. Diagnoses obtained after SPECT MPI included palpitations, anxiety, asthma, myalgia and gastroesophageal reflux disease. In 31 cases (34%), no diagnosis was obtained.

As shown in Table 3, the IL-6, ACTH and cortisol response patterns differed between CAD and non-CAD groups. In all CAD patients, IL-6 increased significantly after exercise while no difference was seen in HPA axis variables. If we included only CAD patients with prior MI (n = 25) a similar pattern was seen, i.e. IL-6 increased from 2.9 (2.4–3.9) to 3.2 (2.6–4.2) pg/mL, p = 0.013) while no significant changes occurred in HPA axis variables. In contrast, the non-CAD patients exhibited significant increases in HPA axis variables but no change in IL-6. FABP4 increased in both CAD and non-CAD groups further supporting that adrenergic responses to the exercise test were similar.

In order to evaluate the influence of sex on IL-6, ACTH and cortisol levels, we compared the 45 males and 43 females in the non-CAD group. Females had lower ACTH and salivary cortisol levels at baseline. However, the IL-6, ACTH and cortisol response patterns were similar in males and females (see Table 1, Supplements).

**Figure 2:**
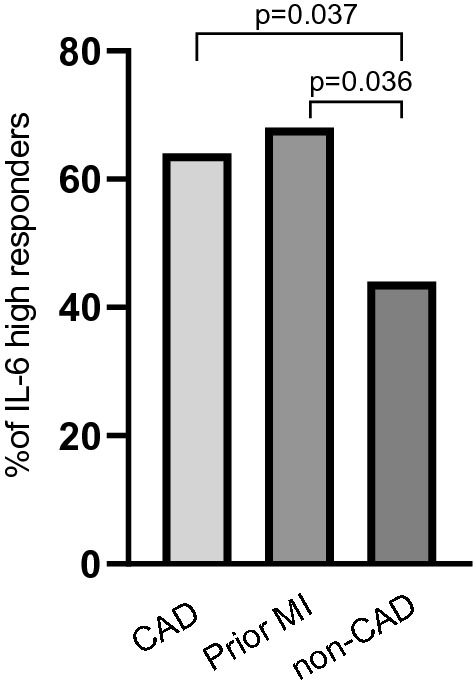
The proportions (%) of IL-6 high responders among patients with documented CAD, defined as a history of prior MI and/or coronary revascularization and/or a myocardial perfusion deficit (n = 36), in patients with a history of prior MI (n = 25) and in patients with no signs of CAD, i.e. no history of prior CAD events and normal SPECT MPI (n = 88).

As an alternative approach, we divided the patients into IL-6 high responders and IL-6 low responders defined as above or below the median value of IL-6 (0.19 pg/mL), see Table 2, Supplements. The proportions of females were similar, 39% and 40%, respectively. As shown in Fig. 2, being a high IL-6 responder was significantly more common among CAD patients, defined as a history of prior MI and/or coronary revascularization and/or a myocardial perfusion deficit, compared with non-CAD patients. The similar result was obtained if only patients with prior MI (n = 25) were included in the analysis. After adjusting for sex, the odds ratio (OR) for documented CAD was 2.03 (p = 0.045) in IL-6 high responders compared with IL-6 low responders. Similarly, using history of prior MI as dependent variable, the OR was 2.70 (p = 0.045) after sex adjustment. If myocardial perfusion deficit was used as dependent variable, the OR for ischemic myocardial perfusion deficit was 1.23 (p = 0.220) in IL-6 high responders compared with IL-6 low responders.

As expected, cortisol levels in plasma and saliva were strongly correlated, p < 0.001, as were ACTH and cortisol in plasma, p < 0.001, whereas IL-6 or CRP levels did not correlate with either ACTH or cortisol levels in the whole study population. However, in the non-CAD group, ACTH and IL-6 levels showed weak but significant inverse correlations at baseline, *r* = -0.211, p = 0.046, and after exercise, *r* = -0.246, p = 0.021. Also, ACTH after exercise correlated inversely with CRP, *r* = -0.267, p = 0.012, in the non-CAD group. There were no correlations between inflammatory markers, ACTH or cortisol in the CAD group. The IL-6, ACTH or cortisol responses did not show any significant correlations with exertion level, left ventricular ejection fraction or degree of myocardial perfusion deficit (data not shown).

We also divided the whole study cohort into subgroups based on medication; statins, beta-blockers, low-dose aspirin or ACE inhibitors/angiotensin receptor blockers, but found no significant differences in IL-6, ACTH and cortisol levels, neither before or after stress (data not shown).

## Discussion

The main findings of the present study indicate an unbalanced relationship between IL-6 and HPA axis variables in patients with documented CAD. A single bout of maximal exercise caused a significant rise in IL-6 but no increase in ACTH and cortisol whereas the opposite pattern was seen in patients without any signs of CAD.

In the whole study cohort, IL-6, ACTH and cortisol increased significantly after exercise. This is in line with previous studies showing that acute exercise is a useful method to investigate stress-induced inflammation. An increase in IL-6 has been reported to be the earliest and most prominent of the cytokine responses to exercise, though strongly related to intensity and duration of the exercise^19,20^. It is also well known that transient elevations in IL-6 induce increased levels of ACTH and cortisol in humans, as part of a normal stress response^12–14^. Thus, for most people, the inflammatory response to acute exercise should be blunted almost immediately by anti-inflammatory mediators stimulated simultaneously by the bout of physical exercise.

However, the IL-6 response to a single bout of maximal exercise varied substantially among study participants, being larger in those with CAD compared to non-CAD despite comparable levels of exercise intensity and duration. The findings agree with previous work reporting that inflammatory response to acute physical exercise, assessed by IL-6 or CRP, is exaggerated in CAD patients compared with healthy controls^10,11^. One plausible explanation of stress-induced inflammatory response in CAD patients could be myocardial ischemia. Two previous studies have examined the inflammatory response to mental stress in relation to inducible myocardial ischemia in CAD patients but found no relationship^21,22^. Neither did we find any evidence that exercise-induced ischemia per se was linked to an exaggerated IL-6 response.

Another striking difference between CAD and non-CAD patients was the lack of increase in ACTH and cortisol in CAD patients after exercise. In line with this finding, a few previous studies have reported that the cortisol response to acute physical or mental stress is blunted in CAD patients compared with healthy subjects^11,23^. Furthermore, there is evidence of other manifestations of HPA axis dysfunctions in patients with CAD, characterized by elevated evening levels and flattening of the diurnal cortisol slope^11,24^. A flattened diurnal cortisol slope before coronary artery bypass graft surgery has been shown to identify those who are at risk of adverse cardiac events and death after surgery^25^. Also, population-based studies have demonstrated that a flattened diurnal cortisol slope is associated with both coronary calcification and increased risk of cardiovascular disease mortality^26,27^.

One intriguing question is whether the lack of ACTH and cortisol response in the CAD group represents a hypoactive HPA axis and thereby a disrupted homeostasis. Several experimental studies have demonstrated an association between abnormally low glucocorticoid response to stress and susceptibility to inflammation^15^. Impaired cortisol response to acute physical exercise has also been described in human chronic inflammatory disorders, such as rheumatoid arthritis and chronic obstructive pulmonary disease^16^. The lack of ACTH response in CAD patients along with the lost association between ACTH and IL-6 levels further highlight the possibility of imbalance between cytokine and cortisol response to stress. Interestingly, Silverman et al.^28^ investigated the role of ACTH in adrenal glucocorticoid response to infection in a mouse model and found that IL-6 failed to elicit glucocorticoid response in the absence of ACTH.

Previous research has described the development of HPA axis hyporeactivity in individuals exposed to chronic stress or suffering from stress-related bodily disorders^29,30^. There is emerging evidence that psychological factors may play a role in the development of HPA axis hypoactivity in CAD. According to Nikkheslat et al.^17^, depressed CAD patients had higher levels of IL-6 gene expression in blood mononuclear cells and lower plasma and saliva cortisol levels compared with non-depressed CAD patients under resting conditions, suggesting that the inflammatory state in depressed CAD patients might be inadequately restrained by endogenous glucocorticoids. In line with this, Waller et al.^23^ reported that depressed CAD patients had a more blunted cortisol response to mental stress than non-depressed CAD patients. In the present study, data on psychosocial stress variables, such as depression, were not available. Only, it was noted that the use of anti-depressive agents was equally low in CAD and non-CAD patients as well as in IL-6 high and low responders.

Another relevant question is whether the increased use of drugs, such as statins or beta blockers, in CAD patients had any suppressive effect on the HPA axis response. As inhibitor of steroid biosynthesis, statins can thereotically influence steroid hormone production. However, levels or rhythms of ACTH and cortisol seem to be unaffected by statin treatment, as recently stated in a Scientific Statement from American Heart Association^31^. Also, the effect of beta blockers on cortisol response to stress has been investigated previously. Compared with placebo, beta blockade resulted in higher cortisol levels after mental stress as well as after short-term maximal dynamic exercise^32,33^. In the present study, neither the use of statins, nor beta blockers seemed to influence the HPA axis response.

Finally, some major limitations of our study should be addressed. Firstly, samples were collected at one single time point 30 min after exercise. This time point was chosen based on data from previous studies using acute bicycle exercise^11,34^ but still, it leaves us unaware of very early transient changes as well as delayed responses. Secondly, the exercise tests took place in the morning between 08 am and 12 noon, raising the possibility that basal cortisol circadian rhythm might influence the cortisol stress response. However, as shown previously^35,36^, there is no evidence of association between cortisol diurnal decline and the magnitude of cortisol stress response. Furthermore, a reanalysis of five independent studies led to the conclusion that the HPA axis response to psychosocial stress was similar in the morning and afternoon^37^. Thirdly, we did not include healthy asymptomatic sex- and age-matched controls. Even if we were able to define non-CAD patients as individuals without a documented history of CAD and a normal SPECT MPI, they were still patients with different types of chest discomfort. The higher proportion of women in the non-CAD group was not an unexpected finding since symptoms of atypical chest pain as well as inconclusive exercise stress tests are known to be more common in women than men^38^. Yet, we found no evidence that the IL-6 or HPA axis stress response differed between men and women,

## Conclusion

By using an exercise test combined with SPECT MPI as a model for stress-induced inflammation, we show that an exaggerated IL-6 response occurred more frequently in patients with documented CAD. The IL-6 response was not related to either exercise intensity and duration or myocardial ischemia. Instead, the findings indicate that the IL-6 response in CAD patients could be out of balance due to HPA axis hypoactivity. Further work needs to be done to establish whether this imbalance is a marker of vulnerability and a potential diagnostic and therapeutic target in CAD.

## Methods

### Patient population

Patients referred for a bicycle ergometry exercise test combined with SPECT MPI were consecutively recruited at the Department of Clinical Physiology, Linköping University Hospital from September 2018 to April 2019. The referral for stress testing was at the clinical discretion of a cardiologist. Subjects were excluded only if they were using oral or parenteral treatment with nonsteroidal anti-inflammatory drugs or any other immunosuppressive agent, such as glucocorticoids or cytostatic agents. Baseline demographic data and medical history were collected retrospectively from medical records, whereas test results were recorded prospectively. The research protocol was approved by the Regional Ethical Review Board in Linköping, Sweden, and informed consent was obtained from all participants. All procedures performed in the study were carried out in accordance with relevant guidelines and regulations that comply with institutional, national, or international gudielines.

### Exercise stress test and sampling procedure

The MPI SPECT tests took place between 08 am and 12 noon. Patients were continued on their daily medication regimen, including anti-ischemic drugs, but were instructed to avoid tooth brushing, smoking, eating and drinking for at least 2 h before the test. The exercise stress test was performed on the electronically braked bicycle Ergometer Ebike Basic Comfort (GE Healthcare, Freiburg, Germany) until exhaustion. The exercise protocol was determined based on estimation of the individual´s capacity, starting at 30–50 W and continuously increased by 10–20 W/min. Heart rate and blood pressure were monitored and recorded before and during each stage of exercise. General exhaustion, anginal chest pain and dyspnea were recorded every minute according to the 20 (general exhaustion and leg fatigue) or 10 (anginal chest pain and dyspnea) graded Borg scale. Continuous 12-lead electrocardiogram (ECG) monitoring was performed before, during and 3–5 min after exercise.

Baseline saliva and blood samples were collected prior to the exercise test (after at least 20 min of rest) and post-exercise saliva and blood samples were collected 30 min after the completion of exercise test. First, saliva was collected with Salimetrics Oral Swabs (Salimetrics via Electrabox, Stockholm, Sweden) placed under the tongue for 2 min. The saliva samples were then immediately placed on ice. Thereafter, whole blood was collected in 9 mL EDTA tubes (BD Biosciences) and plasma was obtained after centrifugation for 10 min at 1500 × g. Centrifugation had to be started within 30 min after blood collection. Saliva and plasma samples were frozen at -70 °C until analysis.

### Myocardial perfusion imaging

MPI was performed according to the European Association of Nuclear Medicine guidelines^39^. At the highest work load and exercise level, technetium-99 m (^99m^Tc) tetrofosmin was injected whereon the patient continued to cycle for 1–2 min. Imaging was performed in upright and supine position with at least 1 million myocardial counts for each imaging session using a semiconductor cadmium-zinc-telluride (CZT) SPECT camera (Spectrum Dynamics, Israel). If the stress imaging studies showed normal perfusion, no additional resting imaging studies were required. In the remaining cases, the patients were asked to return for a resting image two days later. ^99m^Tc tetrofosmin was then administered during rest, followed by SPECT image acquisition after approximately 1 h. Visual analysis was performed by three different nuclear medicine consultants (each with at least 10 years experience in MPI) to assess left ventricular myocardial perfusion during stress and rest and the degree to which the deficit was reversible, according to current guidelines^40^. Ischemic TPD was calculated from the difference between the stress and rest TPD scores using quantitative perfusion software (QPS, version 2012, Cedars-Sinai Medical Center) and the normal databases supplied by the vendour (Spectrum Diagnostic). An abnormal criterion for ischemic TPD was defined as 5% or more^41^. Results close to 5% as well as ambiguous results were assessed and classified by a second nuclear medicine consultant.

Left ventricular ejection fraction was measured by using ECG-gated imaging with 8 frames per cardiac cycle (Quantitative Gated SPECT (QGS) software version 2012, Cedars-Sinai Medical Center). Stress images were used in the analyses.

### Plasma measurements

IL-6 in plasma was analyzed with Luminex Performance Human High Sensitivity Cytokine Panel (Biotechne, Abingdon, UK). FABP4 in plasma was analyzed with Human Premixed Multi-Analyte Kit (Biotechne). The analyses were performed according to the manufacturer’s instructions. The inter-assay coefficients of variation (CV) were 6.4 and 1.0% for IL-6 and FABP4, respectively and the ranges of the standard curves were 0,83 – 3400 pg/mL and 0,61 – 148 ng/mL, respectively. CRP was measured at the Department of Clinical Chemistry at the Linköping University hospital using an immunoturbidimetric assay with a Roche Cobas C502 analyzer (Roche Diagnostics, Scandinavia AB), with a CV of 2.2%.

ACTH and cortisol in plasma were analyzed at the Department of Clinical Chemistry at the Linköping University hospital using an electrochemiluminescence immunoassay with a Roche Cobas e602 (Roche diagnostics) with CV of 1.1 and 3.4%, respectively.

Free cortisol was measured in saliva. After thawing, the samples were centrifuged at 2000 × g for 10 min at 4 °C before 300 µl was mixed with deuterated internal standard D4-cortisol. After extraction with ethyl acetate the organic phase was collected and evaporated under nitrogen. Samples were finally diluted in 10% methanol and then analyzed with liquid chromatography-mass spectrometry (LC–MS/MS)^42^. Four standards with concentrations between 0.625 – 40 nmol/L were used. The intra-assay precision was 6.6% (CV).

### Statistics

IBM SPSS Statistics 25 was used for statistical analyses. Paired sample t-test (for normally distributed data) or Wilcoxon Signed rank test (for non-normally distributed data) were used to compare paired samples. Student´s t-test or Mann–Whitney *U* test were used for comparisons between groups, and Chi square for comparison of categorical data. Correlation analyses were performed by using Spearman ´s rank correlation or logistic regression analysis. Two-tailed p values < 0.05 were considered significant. Numerical data are consistently presented as median (inter-quartile range).

## Supplementary information

Supplementary Information 1.

## Data Availability

The datasets used and analysed during the current study are available from the corresponding author on reasonable request.
