# Supplementary material for: The effect of acute exercise on interleukin-6 and hypothalamic–pituitary–adrenal axis responses in patients with coronary artery disease
Source: Sci Rep. 2020 Dec 7;10:21390. doi: 10.1038/s41598-020-78286-2 (PMC7721799; doi:10.1038/s41598-020-78286-2)
Supplement: Supplementary file 1 — Supplementary Information 1. [file 41598_2020_78286_MOESM1_ESM.docx]

**Supplementary information**

**The effect of acute exercise on interleukin-6 and hypothalamic-pituitary-adrenal axis responses in patients with coronary artery disease.**

Zeid Mahmood, Anette Davidsson, Eva Olsson, Per Leanderson, Anna K Lundberg, Lena Jonasson

**Table 1.** Descriptive data of IL-6 high responders and IL-6 low responders to acute physical exercise, defined as above or below the median value of IL-6 Δ (0.19 pg/mL).

|  | | **IL-6 high responder**  n = 62 | **IL-6 low responder**  n = 62 | p^a^ |
| --- | --- | --- | --- | --- |
| IL-6 Δ, pg/mL | | 0.55 (0.38-0.84) | -0.31 ((-0.60)-0.00) | < 0.001 |
|  | | | | |
| Age, years | | 68 (58-73) | 67 (54-73) | NS |
| Female, n (%) | | 24 (39) | 25 (40) | NS |
| BMI | | 27 (24-30) | 27 (24-28) | NS |
| Smokers, n (%) | | 9 (15) | 6 (9.7) | NS |
| Hypertension, n (%) | | 34 (55) | 45 (45) | NS |
| Diabetes, n (%) | | 10 (16) | 10 (16) | NS |
| CAD, n (%) | | 23 (37) | 13 (21) | 0.037 |
| Prior MI, n (%) | | 17 (27) | 8 (13) | 0.036 |
| Statin, n (%) | | 32 (52) | 26 (42) | NS |
| Beta blockers, n (%) | | 23 (37) | 26 (42) | NS |
| ACEI/ARB, n (%) | | 36 (58) | 25 (40) | 0.035 |
| Calcium channel blockers, n (%) | | 14 (23) | 6 (9.7) | 0.043 |
| Platelet inhibitors, n (%) | | 34 (55) | 21 (34) | 0.015 |
| SSRI, n (%) | | 8 (13) | 5 (8.1) | NS |
| Creatinine, μmol/L | | 82 (68-95) | 82 (68-99) | NS |
| CRP, mg/L | | 0.8 (0.5-2.1) | 1.1 (0.8-2.9) | NS |
|  | | | | |
| Heart rate, beats/min | Baseline | 69 (63-79) | 70 (63-80) | NS |
|  | Maximum | 142 (133-157) | 142 (132-154) | NS |
| Systolic blood pressure, mm Hg | Baseline | 135 (125-145) | 140 (120-150) | NS |
|  | Maximum | 195 (180-210) | 190 (180-205) | NS |
| Diastolic blood pressure, mm Hg | Baseline | 80 (70-85) | 80 (70-90) | NS |
| Maximal workload, watts | | 119 (98-185) | 130 (109-178) | NS |
| Exercise duration, min | | 7.5 (6.4-8.6) | 7.1 (6.0-8.1) | NS |
| Exercise-induced chest pain, n (%) | | 7 (11) | 10 (16) | NS |
| Myocardial perfusion deficit, n (%) | | 12 (19) | 7 (11) | NS |
| Reversible myocardial perfusion deficit, n (%) | | 10 (16) | 4 (6.5) | NS |

^a^IL-6 high responders *vs* IL-6 low responders. BMI, body mass index; CAD, coronary artery disease defined as a history of prior myocardial infarction and/or coronary revascularization and/or a myocardial perfusion deficit. MI, myocardial infarction. ACEI/ARB, angiotensin converting enzyme inhibitors/angiotensin receptor blockers; SSRI, selective serotonin reuptake inhibitor; CRP, C-reactive protein.

**Table 2.** Biochemical measures before and after exercise in male and female non-CAD patients, i.e. no history of prior CAD events and normal SPECT MPI (non-CAD).

|  | **Males**, n = 45 | **Females**, n = 43 | **p** |
| --- | --- | --- | --- |
| *IL-6, pg/mL* | | | |
| Baseline | 2.6 (2.0-4.3) | 3.2 (2.2-4.5) | NS |
| Exercise | 2.5 (2.0-4.6) | 3.2 (2.5-3.8) | NS |
| p^a^ | NS | NS |  |
| *ACTH, pmol/L* | | | |
| Baseline | 3.2 (2.2-4.7) | 2.7 (1.7-4.1) | 0.014 |
| Exercise | 4.2 (2.8-5.2) | 3.1 (2.0-5.4) | NS |
| p^a^ | 0.046 | 0.010 |  |
| *Cortisol in plasma, nmol/L* | | | |
| Baseline | 336 (278-391) | 293 (241-405) | NS |
| Exercise | 353 (313-414) | 359 (263-515) | NS |
| p^a^ | 0.041 | 0.030 |  |
| *Cortisol in saliva, nmol/L* | | | |
| Baseline | 6.7 (4.3-9.3) | 5.3 (3.5-7.6) | 0.021 |
| Exercise | 8.2 (6.2-13) | 8.5 (3.8-15) | NS |
| p^a^ | 0.052 | 0.036 |  |

^a^CAD *vs* non-CAD. ^a^Baseline vs exercise. IL-6, interleukin-6; ACTH, adrenocorticotrophic hormone.
